# 1,1′-[(Biphenyl-4,4′-di­yl)bis­(methyl­ene)]di-1*H*-imidazol-3-ium tetra­chlorido­mercurate(II)

**DOI:** 10.1107/S1600536811047374

**Published:** 2011-11-12

**Authors:** Bo Wen, Guang-Feng Hou, Ying-Hui Yu, Jin-Sheng Gao

**Affiliations:** a College of Chemistry and Materials Science, Heilongjiang University, Harbin 150080, People’s Republic of China; b Engineering Research Center of Pesticides of Heilongjiang University, Heilongjiang University, Harbin 150050, People’s Republic of China

## Abstract

In the title compound, (C_20_H_20_N_4_)[HgCl_4_], the Hg^II^ ion is four-coordinated in a tetra­hedral environment defined by four chloride ions. The dihedral angle between the two phenyl rings is 32.83 (15)°. The protonated 1,1′-[(biphenyl-4,4′-di­yl)bis­(meth­yl­ene)]di-1*H*-imidazol-3-ium cations, showing a *cis* conformation, link the [HgCl_4_]^2−^ anions into an *R*_4_^4^(42) motif *via* N—H⋯Cl hydrogen bonds.

## Related literature

For the synthesis of the ligand, see: Zhu *et al.* (2002 ▶).
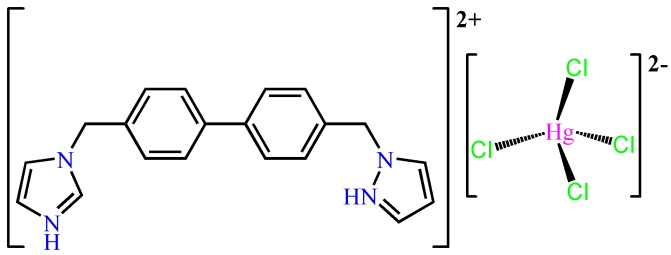


## Experimental

### 

#### Crystal data

(C_20_H_20_N_4_)[HgCl_4_]
*M* *_r_* = 658.79
Monoclinic, 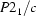

*a* = 8.9318 (18) Å
*b* = 15.347 (3) Å
*c* = 16.840 (3) Å
β = 92.62 (3)°
*V* = 2306.0 (8) Å^3^
*Z* = 4
Mo *K*α radiation
μ = 7.15 mm^−1^
*T* = 293 K
0.24 × 0.23 × 0.22 mm

#### Data collection

Rigaku R-AXIS RAPID diffractometer
Absorption correction: multi-scan (*ABSCOR*; Higashi, 1995 ▶) *T*_min_ = 0.280, *T*_max_ = 0.306
22111 measured reflections
5260 independent reflections
3913 reflections with *I* > 2σ(*I*)
*R*_int_ = 0.046

#### Refinement

*R*[*F* ^2^ > 2σ(*F* ^2^)] = 0.032
*wR*(*F* ^2^) = 0.068
*S* = 1.03
5260 reflections
269 parameters
2 restraints
H atoms treated by a mixture of independent and constrained refinement
Δρ_max_ = 0.98 e Å^−3^
Δρ_min_ = −0.94 e Å^−3^

### 

Data collection: *RAPID-AUTO* (Rigaku, 1998 ▶); cell refinement: *RAPID-AUTO*; data reduction: *CrystalStructure* (Rigaku/MSC, 2002 ▶); program(s) used to solve structure: *SHELXS97* (Sheldrick, 2008 ▶); program(s) used to refine structure: *SHELXL97* (Sheldrick, 2008 ▶); molecular graphics: *DIAMOND* (Brandenburg, 1999 ▶); software used to prepare material for publication: *SHELXTL* (Sheldrick, 2008 ▶).

## Supplementary Material

Crystal structure: contains datablock(s) I, global. DOI: 10.1107/S1600536811047374/hy2485sup1.cif

Structure factors: contains datablock(s) I. DOI: 10.1107/S1600536811047374/hy2485Isup2.hkl

Additional supplementary materials:  crystallographic information; 3D view; checkCIF report

## Figures and Tables

**Table 1:** Hydrogen-bond geometry (Å, °)

*D*—H⋯*A*	*D*—H	H⋯*A*	*D*⋯*A*	*D*—H⋯*A*
N2—H21⋯Cl1^i^	0.90 (1)	2.25 (2)	3.134 (5)	170 (7)
N4—H41⋯Cl3^ii^	0.90 (1)	2.45 (4)	3.168 (5)	137 (5)

Symmetry codes: (i) 
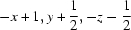
; (ii) 
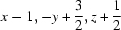
.

**Fig. 1.**
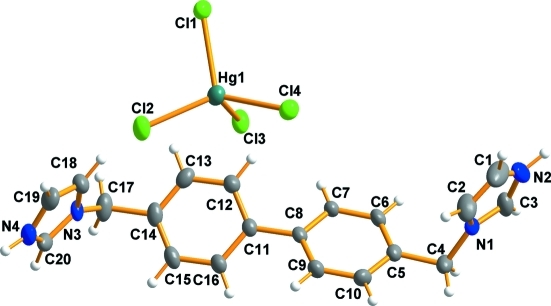
The molecular structure of the title compound, showing displacement ellipsoids at the 50% probability level.

**Fig. 2.**
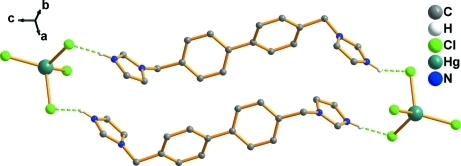
A partial packing view, showing the hydrogen-bonded R44(42) motif.

## supplementary crystallographic information

### Comment

N-containing ligands with an arene center have been widely used as building blocks for constructing inorganic-organic supramolecular architectures. The title compound was synthesized at a low pH value condition, as an unexpected product during the process of preparing the ligand–Hg complex. Herein, we report its structure.

In the title compound, the Hg^II^ ion is four-coordinated in a tetrahedral environment defined by four Cl ions (Fig. 1). The protonated ligand shows a *cis* conformation, which links the [HgCl_4_]^2-^ anions, forming a R_4_^4^(42) motif via N—H···Cl hydrogen bonds (Fig. 2, Table 1).

### Experimental

The 4,4'-(dimethylenebiphenyl)diimidazol ligand was synthesized as the literature method (Zhu *et al.*, 2002). HgCl_2_ (0.140 g, 0.5 mmol) and the ligand (0.160 g, 0.5 mmol) were dissolved in 10 ml ethanol under stirring to get white deposit. 1M HCl solution had been dropped to adjust the pH value until the deposit dissolved. The obtained solution was allowed to stand for several days. Colorless crystals of the title compound were obtained (yield: 28%) as salt-type adducts of the protonated ligand and [HgCl_4_]^2-^ anion.

### Refinement

H atoms bound to C atoms were placed in calculated positions and treated as riding on their parent atoms, with C—H = 0.93 (aromatic) and 0.97 (methylene) Å and with *U*_iso_(H) = 1.2*U*_eq_(C). N-bound H atoms were located in a differece Fourier map and refined with a restraint of N—H = 0.90 (1) Å and *U*_iso_(H) = 1.5*U*_eq_(N).

### Crystal data

**Table:**

(C_20_H_20_N_4_)[HgCl_4_]	*F*(000) = 1264
*M**_r_* = 658.79	*D*_x_ = 1.898 Mg m^−^^3^
Monoclinic, *P*2_1_/*c*	Mo *K*α radiation, λ = 0.71073 Å
Hall symbol: -P 2ybc	Cell parameters from 15195 reflections
*a* = 8.9318 (18) Å	θ = 3.3–27.5°
*b* = 15.347 (3) Å	µ = 7.15 mm^−^^1^
*c* = 16.840 (3) Å	*T* = 293 K
β = 92.62 (3)°	Block, colorless
*V* = 2306.0 (8) Å^3^	0.24 × 0.23 × 0.22 mm
*Z* = 4	

### Data collection

**Table:**

Rigaku R-AXIS RAPID diffractometer	5260 independent reflections
Radiation source: rotation anode	3913 reflections with *I* > 2σ(*I*)
graphite	*R*_int_ = 0.046
ω scan	θ_max_ = 27.5°, θ_min_ = 3.3°
Absorption correction: multi-scan (*ABSCOR*; Higashi, 1995)	*h* = −10→11
*T*_min_ = 0.280, *T*_max_ = 0.306	*k* = −19→18
22111 measured reflections	*l* = −21→21

### Refinement

**Table:**

Refinement on *F*^2^	Secondary atom site location: difference Fourier map
Least-squares matrix: full	Hydrogen site location: inferred from neighbouring sites
*R*[*F*^2^ > 2σ(*F*^2^)] = 0.032	H atoms treated by a mixture of independent and constrained refinement
*wR*(*F*^2^) = 0.068	*w* = 1/[σ^2^(*F*_o_^2^) + (0.0184*P*)^2^ + 2.3484*P*] where *P* = (*F*_o_^2^ + 2*F*_c_^2^)/3
*S* = 1.03	(Δ/σ)_max_ < 0.001
5260 reflections	Δρ_max_ = 0.98 e Å^−^^3^
269 parameters	Δρ_min_ = −0.94 e Å^−^^3^
2 restraints	Extinction correction: *SHELXL97* (Sheldrick, 2008), Fc^*^=kFc[1+0.001xFc^2^λ^3^/sin(2θ)]^-1/4^
Primary atom site location: structure-invariant direct methods	Extinction coefficient: 0.00514 (18)

### Fractional atomic coordinates and isotropic or equivalent isotropic displacement parameters (Å^2^)

**Table:**

	*x*	*y*	*z*	*U*_iso_*/*U*_eq_	
C1	0.1318 (11)	1.0186 (5)	−0.3528 (4)	0.117 (3)	
H1	0.0501	0.9947	−0.3813	0.140*	
C2	0.1321 (6)	1.0519 (4)	−0.2798 (3)	0.0855 (17)	
H2	0.0506	1.0551	−0.2474	0.103*	
C3	0.3553 (6)	1.0636 (3)	−0.3221 (3)	0.0712 (14)	
H3	0.4566	1.0765	−0.3252	0.085*	
C4	0.3225 (6)	1.1204 (3)	−0.1864 (3)	0.0621 (12)	
H4A	0.2678	1.1743	−0.1795	0.074*	
H4B	0.4280	1.1347	−0.1883	0.074*	
C5	0.2996 (5)	1.0618 (3)	−0.1164 (2)	0.0489 (9)	
C6	0.3775 (5)	0.9845 (3)	−0.1082 (2)	0.0494 (10)	
H6	0.4405	0.9672	−0.1478	0.059*	
C7	0.3633 (4)	0.9323 (2)	−0.0421 (2)	0.0433 (9)	
H7	0.4160	0.8801	−0.0379	0.052*	
C8	0.2708 (4)	0.9572 (2)	0.0182 (2)	0.0395 (8)	
C9	0.1902 (5)	1.0336 (3)	0.0087 (2)	0.0517 (10)	
H9	0.1257	1.0506	0.0476	0.062*	
C10	0.2040 (5)	1.0850 (3)	−0.0577 (3)	0.0571 (11)	
H10	0.1482	1.1360	−0.0631	0.068*	
C11	0.2651 (4)	0.9048 (3)	0.0920 (2)	0.0409 (8)	
C12	0.2847 (4)	0.8145 (3)	0.0914 (2)	0.0481 (9)	
H12	0.2986	0.7860	0.0435	0.058*	
C13	0.2837 (4)	0.7670 (3)	0.1610 (3)	0.0543 (11)	
H13	0.2961	0.7069	0.1591	0.065*	
C14	0.2648 (4)	0.8067 (3)	0.2333 (3)	0.0555 (11)	
C15	0.2461 (5)	0.8959 (3)	0.2345 (3)	0.0575 (11)	
H15	0.2340	0.9241	0.2827	0.069*	
C16	0.2449 (4)	0.9439 (3)	0.1652 (2)	0.0507 (10)	
H16	0.2303	1.0039	0.1674	0.061*	
C17	0.2680 (5)	0.7536 (4)	0.3091 (3)	0.0738 (15)	
H17A	0.3061	0.6957	0.2986	0.089*	
H17B	0.3351	0.7809	0.3486	0.089*	
C18	−0.0065 (5)	0.7105 (3)	0.3035 (3)	0.0578 (11)	
H18	−0.0115	0.6846	0.2535	0.069*	
C19	−0.1192 (6)	0.7189 (3)	0.3521 (3)	0.0702 (13)	
H19	−0.2171	0.6996	0.3425	0.084*	
C20	0.0762 (7)	0.7775 (3)	0.4100 (3)	0.0679 (14)	
H20	0.1380	0.8065	0.4472	0.082*	
Cl1	0.69235 (14)	0.46688 (7)	0.05336 (7)	0.0659 (3)	
Cl2	0.58278 (14)	0.62872 (8)	0.22325 (7)	0.0724 (3)	
Cl3	0.94394 (12)	0.67508 (8)	0.09563 (7)	0.0640 (3)	
Cl4	0.58329 (12)	0.71468 (7)	−0.02401 (7)	0.0606 (3)	
Hg1	0.67130 (2)	0.626552 (12)	0.089746 (11)	0.06180 (9)	
N1	0.2722 (4)	1.0801 (2)	−0.26138 (18)	0.0456 (7)	
N2	0.2709 (9)	1.0261 (3)	−0.3772 (3)	0.1021 (19)	
H21	0.294 (8)	1.011 (5)	−0.4267 (18)	0.153*	
N3	0.1169 (4)	0.7466 (2)	0.34045 (19)	0.0512 (8)	
N4	−0.0658 (6)	0.7599 (3)	0.4169 (3)	0.0741 (12)	
H41	−0.116 (6)	0.772 (4)	0.461 (2)	0.111*	

### Atomic displacement parameters (Å^2^)

**Table:**

	*U*^11^	*U*^22^	*U*^33^	*U*^12^	*U*^13^	*U*^23^
C1	0.172 (8)	0.099 (5)	0.073 (5)	−0.059 (5)	−0.052 (5)	0.018 (4)
C2	0.072 (3)	0.117 (5)	0.067 (4)	−0.037 (3)	−0.011 (3)	0.019 (3)
C3	0.084 (3)	0.083 (3)	0.049 (3)	0.012 (3)	0.018 (3)	0.015 (3)
C4	0.088 (3)	0.055 (2)	0.043 (2)	−0.022 (2)	−0.006 (2)	0.005 (2)
C5	0.065 (3)	0.047 (2)	0.035 (2)	−0.010 (2)	−0.0025 (18)	0.0011 (17)
C6	0.058 (2)	0.059 (2)	0.032 (2)	−0.003 (2)	0.0072 (18)	−0.0039 (18)
C7	0.055 (2)	0.0415 (19)	0.033 (2)	0.0007 (18)	0.0055 (17)	−0.0010 (16)
C8	0.0410 (19)	0.047 (2)	0.0307 (19)	−0.0056 (17)	−0.0004 (15)	−0.0031 (16)
C9	0.061 (2)	0.054 (2)	0.040 (2)	0.004 (2)	0.0092 (19)	−0.0026 (19)
C10	0.079 (3)	0.044 (2)	0.049 (3)	0.008 (2)	−0.001 (2)	0.0016 (19)
C11	0.0353 (18)	0.051 (2)	0.036 (2)	−0.0043 (17)	0.0016 (15)	0.0027 (17)
C12	0.048 (2)	0.054 (2)	0.042 (2)	−0.0058 (19)	0.0026 (17)	−0.0002 (19)
C13	0.047 (2)	0.049 (2)	0.066 (3)	−0.0046 (19)	−0.003 (2)	0.015 (2)
C14	0.038 (2)	0.083 (3)	0.046 (3)	−0.003 (2)	0.0025 (18)	0.019 (2)
C15	0.053 (2)	0.084 (3)	0.036 (2)	−0.005 (2)	0.0055 (18)	0.004 (2)
C16	0.054 (2)	0.061 (2)	0.038 (2)	−0.003 (2)	0.0090 (18)	−0.0001 (19)
C17	0.054 (3)	0.104 (4)	0.063 (3)	−0.001 (3)	0.002 (2)	0.037 (3)
C18	0.070 (3)	0.064 (3)	0.039 (2)	−0.009 (2)	0.004 (2)	−0.001 (2)
C19	0.070 (3)	0.072 (3)	0.070 (4)	−0.009 (3)	0.021 (3)	0.015 (3)
C20	0.115 (4)	0.053 (3)	0.035 (2)	−0.015 (3)	−0.012 (3)	0.006 (2)
Cl1	0.0927 (8)	0.0514 (6)	0.0534 (7)	0.0033 (6)	0.0018 (6)	0.0077 (5)
Cl2	0.0760 (7)	0.0845 (8)	0.0583 (7)	0.0128 (7)	0.0199 (6)	0.0173 (6)
Cl3	0.0560 (6)	0.0860 (8)	0.0501 (6)	−0.0079 (6)	0.0026 (5)	0.0116 (6)
Cl4	0.0604 (6)	0.0569 (6)	0.0644 (7)	0.0064 (5)	0.0020 (5)	0.0076 (5)
Hg1	0.06837 (13)	0.05743 (12)	0.06077 (13)	−0.00271 (9)	0.01554 (9)	0.00682 (9)
N1	0.0541 (19)	0.0468 (18)	0.0357 (18)	−0.0079 (16)	0.0010 (15)	0.0042 (14)
N2	0.198 (6)	0.069 (3)	0.038 (3)	0.018 (4)	0.000 (4)	−0.001 (2)
N3	0.060 (2)	0.064 (2)	0.0296 (18)	−0.0103 (17)	0.0025 (15)	0.0118 (16)
N4	0.106 (4)	0.066 (3)	0.053 (3)	0.000 (3)	0.034 (2)	0.011 (2)

### Geometric parameters (Å, °)

**Table:**

C1—N2	1.332 (9)	C12—H12	0.9300
C1—C2	1.332 (9)	C13—C14	1.379 (6)
C1—H1	0.9300	C13—H13	0.9300
C2—N1	1.346 (6)	C14—C15	1.379 (6)
C2—H2	0.9300	C14—C17	1.513 (6)
C3—N2	1.302 (8)	C15—C16	1.381 (6)
C3—N1	1.316 (5)	C15—H15	0.9300
C3—H3	0.9300	C16—H16	0.9300
C4—N1	1.459 (5)	C17—N3	1.476 (5)
C4—C5	1.504 (5)	C17—H17A	0.9700
C4—H4A	0.9700	C17—H17B	0.9700
C4—H4B	0.9700	C18—C19	1.332 (6)
C5—C6	1.379 (6)	C18—N3	1.359 (5)
C5—C10	1.383 (6)	C18—H18	0.9300
C6—C7	1.382 (5)	C19—N4	1.329 (7)
C6—H6	0.9300	C19—H19	0.9300
C7—C8	1.391 (5)	C20—N4	1.307 (7)
C7—H7	0.9300	C20—N3	1.329 (5)
C8—C9	1.382 (5)	C20—H20	0.9300
C8—C11	1.484 (5)	Cl1—Hg1	2.5350 (12)
C9—C10	1.378 (6)	Cl2—Hg1	2.4173 (13)
C9—H9	0.9300	Cl3—Hg1	2.5441 (12)
C10—H10	0.9300	Cl4—Hg1	2.4452 (12)
C11—C16	1.389 (5)	N2—H21	0.90 (1)
C11—C12	1.398 (6)	N4—H41	0.90 (1)
C12—C13	1.380 (5)		
			
N2—C1—C2	106.8 (6)	C15—C14—C13	118.1 (4)
N2—C1—H1	126.6	C15—C14—C17	121.4 (4)
C2—C1—H1	126.6	C13—C14—C17	120.5 (4)
C1—C2—N1	107.4 (6)	C14—C15—C16	120.9 (4)
C1—C2—H2	126.3	C14—C15—H15	119.5
N1—C2—H2	126.3	C16—C15—H15	119.5
N2—C3—N1	108.1 (5)	C15—C16—C11	121.5 (4)
N2—C3—H3	126.0	C15—C16—H16	119.3
N1—C3—H3	126.0	C11—C16—H16	119.3
N1—C4—C5	112.1 (3)	N3—C17—C14	111.0 (4)
N1—C4—H4A	109.2	N3—C17—H17A	109.4
C5—C4—H4A	109.2	C14—C17—H17A	109.4
N1—C4—H4B	109.2	N3—C17—H17B	109.4
C5—C4—H4B	109.2	C14—C17—H17B	109.4
H4A—C4—H4B	107.9	H17A—C17—H17B	108.0
C6—C5—C10	118.4 (4)	C19—C18—N3	107.4 (4)
C6—C5—C4	120.5 (4)	C19—C18—H18	126.3
C10—C5—C4	121.1 (4)	N3—C18—H18	126.3
C5—C6—C7	121.0 (4)	N4—C19—C18	107.3 (5)
C5—C6—H6	119.5	N4—C19—H19	126.3
C7—C6—H6	119.5	C18—C19—H19	126.3
C6—C7—C8	120.6 (4)	N4—C20—N3	108.1 (4)
C6—C7—H7	119.7	N4—C20—H20	126.0
C8—C7—H7	119.7	N3—C20—H20	126.0
C9—C8—C7	118.2 (3)	Cl2—Hg1—Cl4	127.87 (4)
C9—C8—C11	121.3 (3)	Cl2—Hg1—Cl1	105.61 (4)
C7—C8—C11	120.5 (3)	Cl4—Hg1—Cl1	111.72 (4)
C10—C9—C8	121.0 (4)	Cl2—Hg1—Cl3	108.24 (5)
C10—C9—H9	119.5	Cl4—Hg1—Cl3	98.13 (4)
C8—C9—H9	119.5	Cl1—Hg1—Cl3	102.18 (4)
C9—C10—C5	120.9 (4)	C3—N1—C2	108.1 (4)
C9—C10—H10	119.5	C3—N1—C4	126.3 (4)
C5—C10—H10	119.5	C2—N1—C4	125.6 (4)
C16—C11—C12	117.2 (4)	C3—N2—C1	109.7 (5)
C16—C11—C8	121.3 (3)	C3—N2—H21	129 (5)
C12—C11—C8	121.5 (3)	C1—N2—H21	121 (5)
C13—C12—C11	120.7 (4)	C20—N3—C18	107.5 (4)
C13—C12—H12	119.6	C20—N3—C17	125.5 (4)
C11—C12—H12	119.6	C18—N3—C17	127.0 (4)
C14—C13—C12	121.5 (4)	C20—N4—C19	109.7 (4)
C14—C13—H13	119.2	C20—N4—H41	123 (4)
C12—C13—H13	119.2	C19—N4—H41	127 (4)

### Hydrogen-bond geometry (Å, °)

**Table:**

*D*—H···*A*	*D*—H	H···*A*	*D*···*A*	*D*—H···*A*
N2—H21···Cl1^i^	0.90 (1)	2.25 (2)	3.134 (5)	170 (7)
N4—H41···Cl3^ii^	0.90 (1)	2.45 (4)	3.168 (5)	137 (5)

Symmetry codes: (i) −*x*+1, *y*+1/2, −*z*−1/2; (ii) *x*−1, −*y*+3/2, *z*+1/2.
